# Prevalence of Neoplasms in Patients with Acromegaly—A Single-Center Polish Study

**DOI:** 10.3390/jcm13226847

**Published:** 2024-11-14

**Authors:** Martyna Strzelec, Małgorzata Rolla, Justyna Kuliczkowska-Płaksej, Marcin Kałużny, Natalia Słoka, Jakub Wronowicz, Marek Bolanowski, Aleksandra Jawiarczyk-Przybyłowska

**Affiliations:** 1 Department and Clinic of Endocrinology and Internal Medicine, Wroclaw Medical University, Wybrzeże Pasteura 4, 50-367 Wroclaw, Poland; stulamartyna@gmail.com (M.S.);; 2 Statistical Analysis Centre, Wroclaw Medical University, 50-368 Wroclaw, Poland

**Keywords:** acromegaly, pituitary adenoma, neoplasms, IGF-I, GH

## Abstract

**Background/Objectives:** Neoplasms are one of the three most common causes of death in patients with acromegaly. Our study aimed to assess the incidence of benign and malignant neoplasms among patients with acromegaly and the associations between this prevalence and the disease activity, the time of acromegaly diagnosis, and the time of its first symptoms. The correlation between neoplasm occurrence and pituitary somatotropic axis hormone levels was also studied, and the prevalence of different types of neoplasms was compared between the patients with acromegaly and the Polish population. **Methods:** A retrospective study included a statistical analysis of the medical documentation of 230 patients with acromegaly diagnosed and treated in the Department of Endocrinology, Diabetes, and Isotope Therapy in Wrocław (Poland) between 1976 and 2023. **Results:** We observed 171 cases of neoplasms (144 benign and 27 malignant). All types of neoplasms and benign tumors were diagnosed more frequently, in both the short and long term, after a diagnosis of acromegaly, but, after a long time, only malignant neoplasms were more frequently diagnosed. In the cases of controlled acromegaly, all types of neoplasms and benign neoplasms were more common than in cases of cured acromegaly. The incidence of neoplasms was higher, regardless of type, in patients with active acromegaly compared to the cured disease. Malignant neoplasms of the thyroid, renal, and stomach type were more common among our subjects compared to the Polish population. **Conclusions:** This study confirms the association between acromegaly, as well as its activity level and the time from its diagnosis, and the prevalence of neoplasms.

## 1. Introduction

Acromegaly is a rare endocrine disorder caused by excessive secretion of growth hormone (GH), primarily from pituitary tumors (PitNET, pituitary neuroendocrine tumors), resulting in an overproduction of insulin-like growth factor I (IGF-I). The prevalence of this endocrinopathy is estimated at 50–70 cases per million people, with an incidence of 3–4 cases per million population per year. In Poland, over 3000 people suffer from acromegaly [1,2,3]. Most commonly diagnosed in middle-aged individuals, with an average age of 40, it affects female patients in a higher percentage [4,5,6]. From the onset of the first symptoms to the diagnosis of acromegaly and the initiation of effective treatment, the process usually takes about 10 years, although there are new reports showing a reduction in the delay associated with the detection of the disease [3,7,8]. Untreated, acromegaly shortens the expected lifespan by approximately 10 years and leads to numerous complications [9]. Hypersecretion of GH and IGF-I leads to excessive cell proliferation and differentiation, resulting in changes in external appearance and overgrowth of soft tissues, bones, and internal organs, causing various systemic complications such as cardiovascular, metabolic, respiratory, hormonal, and skeletal disorders [10,11,12,13]. The influence of GH and IGF-I excess on neoplasm development has been the subject of numerous studies [10,11,12]. Most likely, these compounds affect the increased proliferation of cells, differentiation, and angiogenesis, partly through the activation of mitogen-activated protein kinases (MAP kinases), dysregulation of the mechanistic target of rapamycin (mTOR) pathway, and modulation of gene transcription in the Janus tyrosine kinase-2/signal transducer and activator of the transcription 3 (JAK-2/STAT-3) signaling pathway. Moreover, they potentially suppress apoptosis through phosphoinositide 3-kinase (PI3 kinase) activity, thereby contributing to tumorigenesis [14,15,16,17,18]. Studies assessing the coexistence of neoplasm with acromegaly provide ambiguous reports. Some researchers have found a higher incidence of thyroid and colorectal cancers [19,20]. Other studies presented a no-higher-than-general-population risk of neoplasm development [21]. According to some researchers, tumors constitute the third-leading cause of death in patients with acromegaly, following cardiovascular and respiratory diseases [7,22,23]. There are also recent studies that show that neoplasms are the leading cause of death [24]. The thyroid gland in patients with acromegaly is structurally and functionally changing. Excess GH and IGF-I induce thyroid proliferation and have anti-apoptotic effects [25]. Available studies have shown that the most common thyroid pathology in patients with acromegaly is goiter; this occurs with a frequency of 20% to 90% (average 55%) in this group of patients. Non-toxic nodular goiter (39.9%) and non-toxic diffuse goiter (17.8%) predominate, while toxic nodular goiter is less common (14.3%) [26].

This single-center study aimed to assess the prevalence of benign and malignant neoplasms in a group of patients with acromegaly. Additionally, we investigated the frequency of goiter in this group of patients.

## 2. Materials and Methods

The analyzed group consisted of 230 patients, 150 women (65%) and 80 men (35%), with acromegaly diagnosed and treated in the Department of Endocrinology, Diabetes, and Isotope Therapy at Wroclaw Medical University (Poland) from 1976 to 2023.

**Figure 1:**
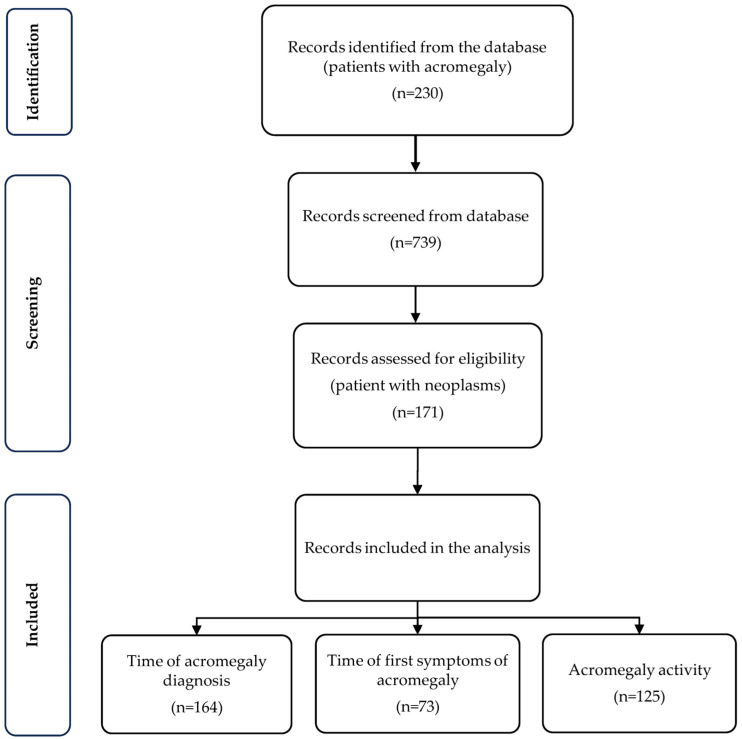
Flow chart of the data included in the analysis.

The inclusion criteria comprised an age at diagnosis of more than 18 years; a diagnosis of acromegaly based on clinical symptoms, as confirmed by the oral glucose tolerance test (OGTT) with GH ≥ 0.4 µg/L; and an elevated IGF-I level according to age and gender, at present or in the past. In patients with diabetes mellitus, OGTT could not be performed. Therefore, in those cases, GH levels were tested five times every 30 min for 2.5 h (fasting and after a meal). Patients who did not fulfil the generally accepted criteria for the diagnosis of acromegaly contained in the Endocrine Society guidelines—elevated IGF-I levels and unsuppressed GH secretion in OGTT at present or in the past—were excluded from the study. The database was created by medical staff working in the clinic, and based on the analysis of 739 medical records from patients with acromegaly and hospitalized in the Department of Endocrinology, Diabetes and Isotope Therapy in Wroclaw (Figure 1). The analysis included demographic data and the frequency, types, and locations of tumors in patients diagnosed with acromegaly. Based on the results of histopathological examinations and the clinical characteristics of the neoplasms, we divided the tumors into categories of benign and malignant. Benign neoplasms were characterized by well-differentiated cells, mostly surrounded by a capsule, without infiltration of neighboring organs or regional lymph nodes, and not giving rise to metastases. Malignant neoplasms included non-encapsulated lesions, with characteristic vascular growth within them, which infiltrated adjacent structures and could give rise to metastases in distant organs. The incidence of particular types of malignancies in our group of patients with acromegaly and in the Polish population was compared; the latter was considered as a theoretical value and calculated on the basis of data from the Polish National Cancer Registry (PNCR) for the years 1999–2021 [27]. For this purpose, the proportionality test was used, and the size of the Polish population was assumed to be the calculated median over the years 1999–2021, which is 38,486,884 persons.

Studies on the frequency of the most common benign tumors (colon) in association with the activity of acromegaly and the level of GH fasting and IGF-I × ULN (Upper Limit of Normal) at the time of cancer diagnosis were also conducted.

Subsequently, the presence of tumors was examined depending on the associated time from the acromegaly diagnosis. Data were divided into three parts, depending on the time of cancer diagnosis: more than the median number of years between acromegaly diagnosis and cancer occurrence (I), less than or equivalent to the median number of years between acromegaly diagnosis and cancer occurrence (II), and before acromegaly diagnosis (III).

Then, the presence of tumors was assessed relative to the origin of the first symptoms of acromegaly. Statistical analyses of tumor occurrence frequency were conducted in patients with acromegaly, divided into three groups based on the timing of tumor diagnosis relative to the appearance of the first symptoms of acromegaly. The division into three groups is analogous to the above analysis of the incidence of neoplasms relative to the acromegaly diagnosis.

Acromegaly activity at the time of cancer diagnosis was also examined. The status of acromegaly was divided into three groups: active acromegaly (AA), where GH ≥ 1.0 µg/L in OGTT and IGF-I was above the age and sex-specific norm; cured acromegaly (CuA), where IGF-I was within the age and sex-specific norm; and controlled acromegaly (CoA), where the constellation of results was similar to that of cured acromegaly during pharmacotherapy.

The associations between the GH 0′, GH 60′, GH 120′ (values from the OGTT test), and IGF-I × ULN concentrations and the occurrence of cancer (all types of cancer, i.e., benign and malignant) were investigated. Correlation analyses of GH fasting and IGF-I concentrations for patients with acromegaly and concomitant cancer in the three groups dependent on disease activity (active, controlled, and cured) versus patients with acromegaly and without cancer, also in these three groups, was performed.

Due to the frequent coexistence of thyroid goiter in patients with acromegaly, the presence of goiter was analyzed relative to acromegaly activity and IGF-I and GH fasting concentrations.

Statistical analyses were performed with Statistica for Windows, version 13.1, by StatSoft (Tulsa, OK, USA). Continuous variables are presented using mean values and standard deviation (SD), and categorical variables as numbers (%); otherwise, the median was used. The following statistical tests were used: the chi-square (χ^2^) test, Fisher test, Shapiro–Wilk test, Kolmogorov–Smirnov test, Mann–Kendall test and Cochran–Mantel–Haenszel test. All *p* values less than 0.05 were considered statistically significant across the whole study. The logistic regression method was used to analyze hormonal results. The odds ratio (OR) parameter was calculated, and the 95% confidence interval was estimated to assess whether certain factors had been associated with tumor development in acromegaly patients.

The research was approved by the Bioethics Committee of Wroclaw Medical University (number: 207/2023). This study was also supported under the Statutory Activities of the Minister of Science and Higher Education (grant number SUBZ.C120.22.020_COVID-19_SNP).

## 3. Results

The study group included 230 patients: 150 women (65%) and 80 men (35%). The clinical characteristics of the study group are presented in Table 1.

### 3.1. The Frequency and Types of Neoplasms, Number of Diagnostic Procedures, and the Prevalence of Goiter in Patients with Acromegaly

In the analyzed group of patients, we recorded 171 neoplasm cases, including 144 (84.2%) benign tumors and 27 (15.8%) malignant tumors. In the female group, we reported a division of neoplasms into 82.8% benign and 17.2% malignant. Meanwhile, in the male group, these were 87.8% (*p* = 0.308) and 12.2% (*p* = 0.178), respectively. The occurrence of more than one tumor in a single patient was observed in 44 patients, with 35 cases in women and 9 in men (*p* = 0.061). The mean age of neoplasm diagnosis for patients with acromegaly was 58.96 ± 12.22 years for women and 53.09 ± 10.4 for men. The cancer categorizations according to location and malignancy are presented in Table 2.

**Figure 2:**
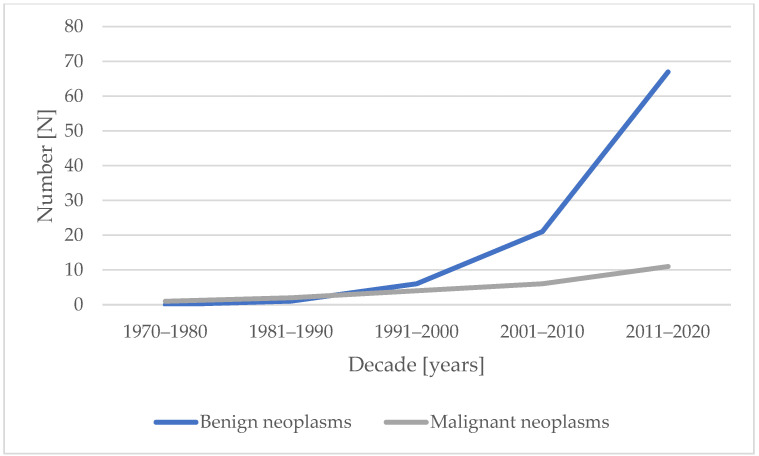
Number of neoplasms in patients with acromegaly over the decades.

Over recent decades, benign and malignant neoplasms have increased among acromegaly patients (Figure 2). In the years 2021–2023, twenty-nine benign neoplasms and four malignant neoplasms were diagnosed.

**Figure 3:**
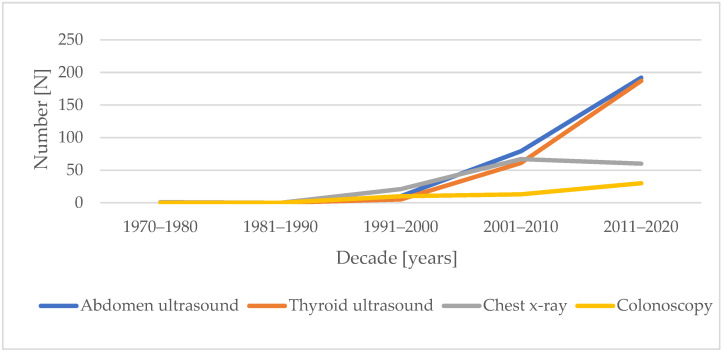
Number of procedures performed on patients with acromegaly over the decades.

We investigated the frequency of diagnostic procedures performed upon or ordered for the patients with acromegaly in our study during hospitalization in the Endocrinology Department. These values are listed in Table 3. The number of abdominal and thyroid ultrasounds performed on subjects has shown a high upward trend over the years, with a lesser increase in the number of colonoscopies (Figure 3). The number of chest X-Rays increased until 2010, and then declined slightly. In the decade just begun (2021–2023), 77 thyroid ultrasounds, 74 abdominal ultrasounds, 37 chest X-Rays, and 12 colonoscopies have been performed. Over the years, the increasing number of diagnostic tests performed did not significantly affect the increase in the number of neoplasms (benign, malignant, or taken together).

**Figure 4:**
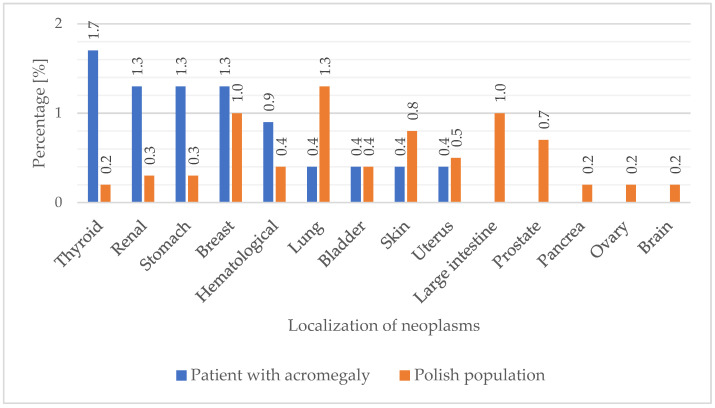
Prevalence of neoplasms according to location, in patients with acromegaly and in the Polish population.

A comparison of the prevalence of malignant neoplasms among patients with acromegaly relative to incidence in the Polish population from 1999 to 2021 is presented in Figure 4. The incidence values for malignant tumors of the thyroid (*p* < 0.000001), renal (*p* = 0.002), and stomach (*p* = 0.005) type were found to be significantly higher among patients with acromegaly, compared to the Polish population.

The analysis showed no correlation between the incidence of benign colorectal tumors and the activity of the acromegaly at the time of neoplasm diagnosis (*p* = 0.166). The study also reported no significant association between the occurrence of these neoplasms and IGF-I × ULN and GH fasting concentrations.

In the examined group, 122 acromegaly patients were diagnosed with goiter in a thyroid ultrasound examination, representing 53% of the study participants (N = 85, 56.7% of women, and N = 37, 46.3% of men). The analysis results concerning the occurrence of thyroid goiter in association with acromegaly activity showed a significantly more frequent incidence of goiter in patients with cured acromegaly than seen in patients with controlled (*p* = 0.0014) and active (*p* < 0.001) forms of the disease. The analysis showed no correlation between the subjects’ parameters, such as GH fasting levels and IGF-I × ULN at the time of acromegaly diagnosis, and the incidence of thyroid goiter.

### 3.2. The Occurrence of Neoplasms Based on the Time of Acromegaly Diagnosis and the Time of First Acromegaly Symptoms

**Figure 5:**
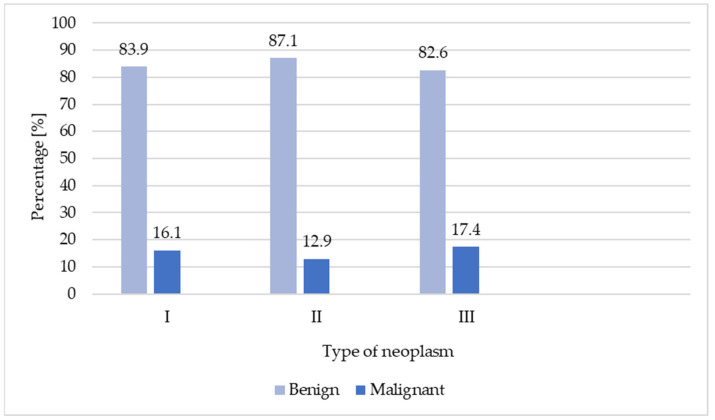
The dependence of neoplasm frequency on the time from acromegaly diagnosis.

The diagnosis of acromegaly significantly influences the likelihood of developing all kinds of neoplasms (II vs. III, *p* < 0.001, and I vs. III, *p* < 0.001) and benign tumors (II vs. III, *p* < 0.001; I vs. III, *p* < 0.001), in both the short and the long term after diagnosis. However, the time elapsed from the diagnosis of acromegaly does not have a significant impact on the occurrence of cancer (I vs. II). The analysis showed a significantly higher incidence of malignant neoplasms occurring after a long period subsequent to acromegaly diagnosis in relation to those with a shorter period of time before the diagnosis (I vs. III, *p* = 0.0176). There was no significant association between the relative time of onset of acromegaly symptoms and the benign or malignant nature of the neoplasms. These results are presented in Figure 5.

### 3.3. The Activity of Acromegaly at the Time of Tumor Diagnosis and the Association Between the Incidence of Neoplasms and the Results of Hormonal Tests

**Figure 6:**
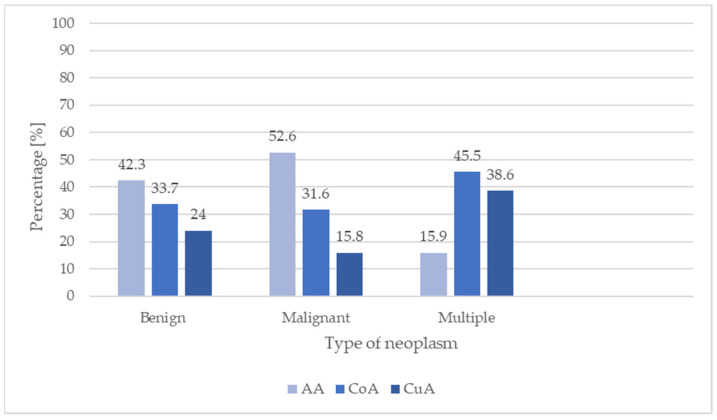
Number of neoplasms in relation to acromegaly activity.

In the controlled acromegaly, all types of neoplasms and benign neoplasms were significantly more frequent, compared to cured acromegaly (CoA vs. CuA, *p* < 0.001 and *p* = 0.0016, respectively). Comparing active and cured acromegaly, we noticed that the incidence of neoplasms was significantly higher, regardless of type (AA vs. CuA, *p* = 0.019) in patients with active acromegaly, while in the case of benign neoplasms, such an association was not observed. The study showed no differences between the incidence of tumors in patients with active acromegaly compared to patients with controlled acromegaly. Based on the analysis of acromegaly activity at the time of malignant tumor diagnosis, there were no significant differences between the groups (*p* = 0.0678). Moreover, multiple neoplasms were significantly more frequent in controlled acromegaly than in active acromegaly (CoA vs. AA *p* = 0.0012). The study showed no significant differences in the incidence of multiple tumors when comparing groups based on acromegaly activity (*p* > 0.05). These results are presented in Figure 6. There is no significant effect of acromegaly activity on the relationship between cancer incidence and the time of acromegaly diagnosis.

There were no significant associations in the between the incidence of benign tumors, malignancies, and both types and the GH 0′, GH 60′, GH 120′, and IGF-I × ULN concentrations. We found no significant differences in the correlation analysis of IGF-I and GH fasting concentrations among patients with concurrent neoplasm in the three acromegaly activity-dependent groups (active, controlled, and cured) or among patients without neoplasm.

## 4. Discussion

Neoplasm occurrence in patients with acromegaly is one of the most frequent factors leading to a deterioration in the quality of life and shortening of life expectancy [28,29,30]. For a long time, the literature [31,32] has mentioned the potential predisposition of acromegaly to tumorigenesis, but research findings have not been consistent on this topic. According to Orme et al., the overall cancer incidence rate was lower than in the general population, and there was no significant increase in the frequency of site-specific cancer occurrences [33]. Furthermore, Holdaway et al. did not observe a significant increase in mortality of acromegaly patients due to tumors. However, individuals with elevated levels of GH or IGF-I had significantly higher cancer-related mortality than those with normal IGF-I or levels of GH lower than 2 µg/L [34]. In our analysis, we found a more frequent occurrence of malignant tumors of the thyroid, renal and stomach types, compared to the general Polish population. The results obtained may be due to the endocrine profile of the patients in our department and the relatively high number of thyroid and abdominal ultrasound examinations recommended for the patients in our study. Current scientific reports indicate a higher incidence of cancer in patients with acromegaly, compared to the general population. In a comparison of the standardized incidence ratio (SIR) of cancer in patients with acromegaly relative to that of the general population, there was an increase in the SIR in the U.S., Swedish, Danish, Finnish, Italian, and Chinese cohorts, with no increase seen in the U.K. and France, and a slightly lower increase found in Germany [14,21,23,35,36,37,38]. Advancements in medical diagnostics and new therapeutic options have led to extensions of the lifespans of acromegaly patients and an earlier diagnosis of acromegaly-related diseases, including tumor formations [39]. This is confirmed by Demir et al., who compared changes in the presentation of acromegaly over half a century. Their study found that patients diagnosed before 2014 had a longer duration of symptoms before diagnosis, larger pituitary adenomas, a higher incidence of cavernous sinus infiltration, higher levels of GH and IGF-I, and a more frequent incidence of comorbidities such as colorectal polyps and thyroid cancer at diagnosis, in comparison to those diagnosed in the last decade [40].

In the population of the patients with acromegaly in our study, we observed a definite female predominance and an age of diagnosis of acromegaly typically in the fifth decade of life. Similar reports are presented by other studies [2,41,42,43]. Benign neoplasms were more frequent in men. An inverse dependency was observed in malignant neoplasms. Multiple neoplasms were more strongly associated with women. We have indicated an upward trend in the incidence of benign and malignant neoplasms among acromegaly patients over the decades. We observed a similar relationship for most diagnostic procedures performed or recommended for the patients with acromegaly in our study. This is most likely due to improved diagnostic methods, easier access to them, greater awareness of oncological complications among doctors, and their more active search. The reason for the relatively low number of colonoscopies performed was most likely the profile of our clinic and the required presence of other specialists during this examination. After 2010, only the number of chest X-Ray examinations declined (slightly), which may be due to the fact that patients have these examinations performed regularly as part of occupational health check-ups. The increasing number of examinations did not significantly affect the increase in the number of cancers (benign, malignant, or taken together). There are fewer recent new cases of benign and malignant neoplasms, compared to the previous decade, because the present decade is not finished and there was limited access to health services during the COVID-19 pandemic within this period.

Predominantly, the benign neoplasms in our group of patients included colorectal (29.17%), uterine (12.5%), liver (12.5%), and adrenal tumors (10.42%). Similarly, more than one-third of the acromegaly population in Can et al. [44] was diagnosed with colon polyps. In Jenkins et al. [45], one in four patients had benign bowel changes. According to studies, the incidence of colon polyps in patients with acromegaly ranged from 6 to 30% for both adenomatous and non-adenomatous lesions, while for colorectal cancer, it ranged from 4 to 10% [46]. Moreover, Rokkas’ results indicated that patients with acromegaly are significantly more likely to develop colorectal adenoma, hyperplastic polyps, or colorectal cancer, compared to the control group [20]. Similar to Can et al., in our study [44], we also reported no significant association between the incidence of benign colorectal tumors and the IGF-I × ULN, GH fasting levels, and acromegaly activity at the time of tumor diagnosis. Contrary to studies with worldwide reporting, these results may be due to the heterogeneous group of patients examined. However, the association of acromegaly with the development of colorectal cancer has been confirmed in numerous studies [7,37,47,48]. Excess GH causes the development of colon polyps and likely also creates a predisposition to the transformation of precancerous lesions into cancer [28,49]. Effective control of acromegaly reduces the mortality of these patients to the rates seen in the general population [50]. Researchers agree on the necessity of screenings for colorectal cancer in acromegaly patients, but the frequency of their performance has not been established [51]. It is worth emphasizing that endoscopic examination of the large intestine is technically difficult to perform in patients with acromegaly and requires longer cecum intubation, compared to the control group [20,47]. This is due to the increased length and capacity of the acromegalic colon and prolonged intestinal transit time [23]. Rigorous bowel preparation for examination is, therefore, crucial. According to Bałdys–Waligórska et al., the most frequent benign neoplasms, apart from those mentioned above, include thyroid neoplasms, as well as those of the prostate, according to Plotuna et al. [16,41]. According to a single-center Polish study, thyroid lesions and multinodular goiter turned out to be significantly more frequent in acromegaly patients, compared to the control group. Thyroid cancer was also dominant, but the difference was not significant [52]. In our analysis, thyroid goiter was not categorized as benign thyroid neoplasms. A separate statistical analysis of this group of patients was performed. The study of the data of the patients in our study showed a significantly higher incidence of goiter in the cured patients than in active and controlled cases. This may result from diagnostics toward other endocrinopathies and complications of acromegaly at a later therapeutic stage. In the literature, we have found studies comparing the association between the acromegaly activity and the volume and change in the nature of thyroid nodules. Kan et al. showed that total thyroid-nodule volume decreased significantly in patients with well-controlled acromegaly, remained unchanged in controlled patients, and increased in patients with active disease [53]. Furthermore, Xu et al. presented a significant change in the thyroid-nodule morphology, from solid lesions in patients before surgery to cystic ones in the group of cured patients and a reduced number of vascular and heterogeneous lesions [54]. In the group of untreated patients, this correlation was not found. Contrary to our analysis, Wu et al. showed that elevated levels of IGF-I and GH are positively correlated with thyroid volume [55]. Furthermore, Dogansen et al. reported a significantly more frequent incidence and growth of thyroid nodules in active acromegaly [56].

The prevalence of malignancy in patients with acromegaly has been reported to be near the range of 6.3% to 14% [11,48]. Our study found the malignancy prevalence to be 11.7%, which was similar to previous studies. Among our acromegaly patients, we observed six cases of breast cancer, four malignant thyroid neoplasms, four hematologic neoplasms, three renal and stomach cancers, and two cases of uterine cancer. In addition, we found isolated cases of cancers of the lung, testicular, bladder, nervous system, and melanoma. We did not observe any cases of colorectal cancer among the patients in our study. The relatively high number of benign neoplastic lesions and the lack of colorectal malignancies among the patients in our study may be due to the strong recommendation of prophylactic colonoscopy and removal of polyps and other benign lesions at an early stage of carcinogenesis. Moreover, this diagnosis could be underestimated in the group of patients in our study due to the lack of an active search for neoplasms during the analyzed hospitalizations. Akhanli et al. showed no differences in the incidence of benign and malignant changes in the mammary glands in comparison to acromegaly patients with controls. Also, no correlation between the incidence of neoplasms and the activity of acromegaly was found. However, higher breast density was found in patients with acromegaly, compared to the control group, as well as a relationship between higher GH concentrations and an increase in the frequency of masses in breast ultrasound examinations [57]. Therefore, the creation of a protocol for breast cancer-preventive examinations among patients with acromegaly is worth considering. Among the histopathological types of thyroid cancer, we noticed two cases of papillary carcinoma and one case of medullary and follicular carcinomas. We have observed a relatively high number of cases of malignant thyroid neoplasms in the patients in our study, which is probably the result of an easily accessible ultrasound examination frequently performed in our clinic. In numerous retrospective studies, the incidence of malignant thyroid neoplasms ranged from 0.8% to 7.2% [41,48,58,59]. However, Terzolo emphasizes that in Italy and other European countries, a significantly higher incidence of thyroid cancer was found in patients with acromegaly and thyroid nodules compared to the numbers for patients with thyroid nodules in the general population, while in North American studies, the incidence was similar [19,60]. It was indicated that this country-related variability may result from different levels of iodine intake in the diet and various uses of ultrasound in diagnostics. Therefore, it is crucial to repetitively control the morphology of the thyroid gland in an ultrasound examination and perform a fine-needle biopsy when necessary in patients with acromegaly.

Our study compared the incidence of neoplasms between categories depending on the time of acromegaly diagnosis. We found a significantly higher incidence of all kinds of neoplasms and benign lesions in patients with acromegaly, for both short and long periods after the diagnosis of the underlying disease, compared to the period before acromegaly diagnosis. The above result indirectly indicates acromegaly to be an important factor in carcinogenesis and emphasizes the importance of active cancer screening from the very beginning of acromegaly diagnosis. Furthermore, we reported a significantly higher incidence of malignant neoplasms diagnosed a long time after acromegaly diagnosis, emphasizing the importance of long-term follow-up for patients with acromegaly, and the value in maintaining oncological vigilance, even at a later stage of the underlying disease. Similarly, one Polish retrospective study found a significant correlation between the number of malignancies and the duration of uncontrolled disease, comparing groups in which this period lasted less than 5 years and for those with over 5 years. Moreover, there was no difference in the number of malignant neoplasms in relation to the total duration of acromegaly (less than 10 years and over 10 years) [41]. Durmuş et al. indicated that the incidence of cancer increases with acromegaly, and that this increase is associated with disease duration [61]. In contrast, Terzolo et al. found no significant association with acromegaly duration before cancer diagnosis [42].

Our research results indicate that the association between the prevalence of neoplasms and the time from the onset of the first acromegaly symptoms was not significant. This is likely because acromegaly begins with varying severity of its symptoms, and the timing of medical admission differs among patients. Similar results were obtained by Plotuna et al. [16].

The next part of our analysis concerned the impact of acromegaly activity on the incidence of neoplasms. We noticed a significantly higher incidence of cancers in a broad sense as well as benign neoplasms in controlled acromegaly compared to cured acromegaly, and neoplasms, regardless of type, in active acromegaly compared to the cured form. The above findings show how important it is to achieve the status of cured acromegaly in the context of oncological events. In contrast to the single-center Polish [41] and Romanian [16] studies, we found no significant difference in neoplasm occurrence in active and controlled acromegaly. This may be due to the discrepancy in the duration of active acromegaly. Interestingly, in our study, multiple neoplasms were significantly more frequent in controlled acromegaly than in the active form. This could be explained by the longer observation period of a patient with controlled disease, in relation to active acromegaly. Furthermore, we observed two cases of acromegaly patients with more than three malignancies. Such persons require searching for a genetic predisposition to neoplasms. A complete genetic testing panel was performed for one patient, but no underlying cause for several cancers was found. It is worth noting that acromegaly may be a component of multiple endocrine neoplasia type 1 (MEN 1), MEN 4, McCune–Albright syndrome, and Carney complex [23], so special vigilance should be exercised in the diagnosis of other tumors.

In our research, no association was found between the levels of pituitary somatotropic axis hormones and the incidence of cancer. There was also no correlation between the levels of these hormones in patients with coexisting neoplasm, compared to patients without tumors, depending on the activity of the acromegaly. Such results are likely due to the heterogeneous group of patients studied and changes in standards, units, and hormone diagnostic methods over the decades. Similarly, Plotuna et al. reported no significant association between the duration of acromegaly, the level of IGF-I and GH, and the risk of malignancy, which was explained by the small group of patients analyzed [16]. Terzolo et al. and Bałdys-Waligórska et al. showed no evidence of a link between these hormones and cancer incidence [41,42]. It was emphasized that the hormonal assessment at the time of cancer diagnosis or at last observation poorly reflects the chronic exposure of the acromegaly patient to GH and IGF-I. Xiao et al. also showed no association between IGF-I and GH levels in a cohort of patients with acromegaly and coexisting neoplasm, compared to patients without cancer. However, in the case-control section, patients with cancer had higher levels of GH, IGF-I, and frequency of uncontrolled disease at the last visit [38].

In conclusion, this study emphasized that neoplasms are significant complications among patients with acromegaly. It is recommended that active screening be performed, starting from the disease diagnosis. Studies on this subject are inconsistent. This is possibly caused by the fact that acromegaly is a rare disease, and the patients are heterogeneous. It is worth emphasizing that in patients with multiple cancers, a genetic predisposition to neoplasm should be suspected, and acromegaly may be part of multiple endocrine neoplasia syndrome. Therefore, vigilance in the diagnosis of other tumors is important. In our retrospective study, some limitations could be found. First, some neoplasms occurred in cases in which active diagnostics were not conducted to detect them. Furthermore, the discipline of the patients in our study diagnosed in the past was relatively low, and some subjects were not followed up with. Therefore, the research needs to be continued in the future. Nevertheless, our findings shed light on the collective occurrence of acromegaly and neoplasms. Further, more specific, prospective studies should be performed to develop a holistic understanding of the topic.

## Figures and Tables

**Table 1:** Clinical characteristics of patients diagnosed with acromegaly.

Characteristics	Women (*n* = 150)		Men (*n* = 80)		*p*
	Mean ± SD	Median	Mean ± SD	Median	
BMI (kg/m^2^)	29.65 ± 5.79	28.57	28.37 ± 3.40	28.08	0.295
Age at diagnosis (years)	49.24 ± 14.95	50.00	43.24 ± 13.09	41.50	0.007
Age at neoplasm diagnosis (years)	58.96 ± 12.22	59.00	53.09 ± 10.4	51.00	0.005
Recurrence of acromegaly, *n* (%)	55 (59.78)		37 (40.22)		0.158
Benign neoplasms, *n* (%)	101 (82.79)		43 (87.76)		0.308
Malignant neoplasms, *n* (%)	21 (17.21)		6 (12.24)		0.178
Multiple neoplasms, *n* (%)	35 (28.69)		9 (18.37)		0.061

**Table 2:** Number of malignant and benign neoplasms in patients with acromegaly, *n* = 171.

Localization	Benign		Malignant		Total, *n* (%)
	Women	Men	Women	Men	
Colon	25	17	-	-	42 (24.56)
Uterus	18	-	2	-	20 (11.7)
Liver	12	6	-	-	18 (10.53)
Adrenal glands	10	5	-	-	15 (8.77)
Central nervous system	7	3	1	-	11 (6.43)
Breast	6	1	6	-	13 (7.6)
Mesenchymal tissue (skin, muscles, bones, lipoma)	6	-	-	1	7 (4.09)
Gallbladder	2	4	-	-	6 (3.51)
Nasopharynx	5	-	-	-	5 (2.92)
Parathyroid glands	4	1	-	-	5 (2.92)
Renal	2	2	2	1	7 (4.09)
Prostate	-	2	-	-	2 (1.17)
Pancreas	2	-	-	-	2 (1.17)
Ovaries	2	-	-	-	2 (1.17)
Stomach	-	1	2	1	4 (2.34)
Salivary gland	-	1	-	-	1 (0.58)
Thyroid	-	-	3	1	4 (2.34)
Hematological	-	-	4	-	4 (2.34)
Lung	-	-	-	1	1 (0.58)
Testis	-		-	1	1 (0.58)
Bladder	-	-	-	1	1 (0.58)
Total	101	43	20	7	171

**Table 3:** Number of procedures performed in patients with acromegaly.

Type of Procedure	Number of Procedures/739 Hospitalizations	%
Abdomen ultrasound	355	48.04
Thyroid ultrasound	330	44.65
Chest X-Ray	186	25.17
Colonoscopy	65	8.8

## Data Availability

The original contributions presented in the study are included in the article; further inquiries can be directed to the corresponding author.

## References

[1] Bolanowski M., Ruchała M., Zgliczyński W., Kos-Kudła B., Hubalewska-Dydejczyk A., Lewiński A. (2019). Diagnostics and treatment of acromegaly—Updated recommendations of the Polish Society of Endocrinology. Endokrynol. Pol..

[2] Matyjaszek-Matuszek B., Obel E., Lewicki M., Kowalczyk-Bołtuć J., Smoleń A. (2018). Prevalence of neoplasms in patients with acromegaly– the need for a national registry. Ann. Agric. Environ. Med..

[3] Chanson P., Salenave S. (2008). Acromegaly. Orphanet J. Rare Dis..

[4] Kamusheva M., Vandeva S., Mitov K., Rusenova Y., Elenkova A., Zacharieva S., Mitkova Z., Tachkov K., Dimitrova M., Doneva M. (2020). New Epidemiological, Clinical and Economic Data for Patients with Acromegaly in Bulgaria. Front. Public Health.

[5] Bolanowski M., Zgliczyński W., Sowiński J., Bałdys-Waligórska A., Bednarek-Tupikowska G., Witek P., Zieliński G., Liebert W., Siemińska L., Andrysiak-Mamos E. (2020). Therapeutic effect of presurgical treatment with longacting octreotide (Sandostatin^®^ LAR^®^) in patients with acromegaly. Endokrynol. Pol..

[6] Petrossians P., Daly A.F., Natchev E., Maione L., Blijdorp K., Sahnoun-Fathallah M., Auriemma R., Diallo A.M., Hulting A.-L., Ferone D. (2017). Acromegaly at diagnosis in 3173 patients from the Liège Acromegaly Survey (LAS) Database. Endocr. Relat. Cancer.

[7] Abreu A., Tovar A.P., Castellanos R., Valenzuela A., Giraldo C.M.G., Pinedo A.C., Guerrero D.P., Barrera C.A.B., Franco H.I., Ribeiro-Oliveira A. (2016). Challenges in the diagnosis and management of acromegaly: A focus on comorbidities. Pituitary.

[8] Slagboom T.N.A., van Bunderen C.C., De Vries R., Bisschop P.H., Drent M.L. (2023). Prevalence of clinical signs, symptoms and comorbidities at diagnosis of acromegaly: A systematic review in accordance with PRISMA guidelines. Pituitary.

[9] Ruchała M., Szczepanek-Parulska E., Fularz M., Woliński K. (2012). Risk of neoplasms in acromegaly. Contemp. Oncol..

[10] Rolla M., Jawiarczyk-Przybyłowska A., Halupczok-Żyła J., Kałużny M., Konopka B.M., Błoniecka I., Zieliński G., Bolanowski M. (2021). Complications and Comorbidities of Acromegaly—Retrospective Study in Polish Center. Front. Endocrinol..

[11] Tseng F.Y., Huang T.S., Lin J.D., Chen S.T., Wang P.W., Chen J.F., Huey-Herng Sheu W., Chang T.C. (2019). A registry of acromegaly patients and one year following up in Taiwan. J. Formos. Med. Assoc..

[12] Colao A., Grasso L.F.S., Di Cera M., Thompson-Leduc P., Cheng W.Y., Cheung H.C., Duh M.S., Neary M.P., Pedroncelli A.M., Maamari R. (2020). Association between biochemical control and comorbidities in patients with acromegaly: An Italian longitudinal retrospective chart review study. J. Endocrinol. Investig..

[13] Tomasik A., Stelmachowska-Banaś M., Maksymowicz M., Czajka-Oraniec I., Raczkiewicz D., Zieliński G., Kunicki J., Zgliczyński W. (2022). Clinical, hormonal and pathomorphological markers of somatotroph pituitary neuroendocrine tumors predicting the treatment outcome in acromegaly. Front. Endocrinol..

[14] Boguszewski C.L., Boguszewski M.C.D.S. (2019). Growth hormone’s links to cancer. Endocr. Rev..

[15] Boguszewski C.L., Boguszewski M.C.D.S., Kopchick J.J. (2016). Growth hormone, insulin-like growth factor system, and carcinogenesis. Endokrynol. Pol..

[16] Plotuna I.S., Balas M., Golu I., Amzar D., Vlad A., Moleriu L.C., Vlad M. (2023). The Experience of a Single Tertiary Center Regarding Benign and Malignant Tumors in Acromegalic Patients. Medicina.

[17] Melmed S. (2009). Acromegaly pathogenesis and treatment. J. Clin. Investig..

[18] Strous G.J., Almeida A.D.S., Putters J., Schantl J., Sedek M., Slotman J.A., Nespital T., Hassink G.C., Mol J.A. (2020). Growth Hormone Receptor Regulation in Cancer and Chronic Diseases. Front. Endocrinol..

[19] Woliński K., Czarnywojtek A., Ruchala M. (2014). Risk of thyroid nodular disease and thyroid cancer in patients with acromegaly—Meta-analysis and systematic review. PLoS ONE.

[20] Rokkas T., Pistiolas D., Sechopoulos P., Margantinis G., Koukoulis G. (2008). Risk of colorectal neoplasm in patients with acromegaly: A meta-analysis. World J. Gastroenterol..

[21] Petroff D., Tönjes A., Grussendorf M., Droste M., Dimopoulou C., Stalla G., Jaursch-Hancke C., Mai M., Schopohl J., Schöfl C. (2015). The incidence of cancer among acromegaly patients: Results from the German acromegaly registry. J. Clin. Endocrinol. Metab..

[22] Bach L.A. (2004). The insulin-like growth factor system: Towards clinical applications. Clin. Biochem. Rev..

[23] Boguszewski C.L., Ayuk J. (2016). Acromegaly and cancer: An old debate revisited. Eur. J. Endocrinol..

[24] Bolfi F., Neves A.F., Boguszewski C.L., Nunes-Nogueira V.S. (2018). Mortality in acromegaly decreased in the last decade: A systematic review and meta-analysis. Eur. J. Endocrinol..

[25] Danilowicz K., Sosa S., Gonzalez Pernas M.S., Bamberger E., Diez S.M., Fainstein-Day P., Furioso A., Glerean M., Guitelman M., Katz D. (2020). Acromegaly and thyroid cancer: Analysis of evolution in a series of patients. Clin. Diabetes Endocrinol..

[26] Dąbrowska A.M., Tarach J.S., Kurowska M., Nowakowski A. (2014). State of the art paper Thyroid diseases in patients with acromegaly. Arch. Med. Sci..

[27] https://onkologia.org.pl/en/report.

[28] Colao A., Ferone D., Marzullo P., Lombardi G. (2004). Systemic Complications of Acromegaly: Epidemiology, Pathogenesis, and Management. Endocr. Rev..

[29] Sheppard M.C. (2004). Growth hormone--from molecule to mortality. Clin. Med..

[30] Kasuki L., Maia B., Gadelha M.R. (2022). Acromegaly and Colorectal Neoplasm: An Update. Front. Endocrinol..

[31] Woliński K., Stangierski A., Dyrda K., Nowicka K., Pelka M., Iqbal A., Car A., Lazizi M., Bednarek N., Czarnywojtek A. (2017). Risk of malignant neoplasms in acromegaly: A case–control study. J. Endocrinol. Investig..

[32] Ruchała M., Woliński K. (2019). Health-Related Complications of Acromegaly—Risk of Malignant Neoplasms. Front. Endocrinol..

[33] Orme S.M., McNally R.J.Q., Cartwright R.A., Belchetz P.E. (1998). Mortality and Cancer Incidence in Acromegaly: A Retrospective Cohort Study 1. J. Clin. Endocrinol. Metab..

[34] Holdaway I.M., Rajasoorya R.C., Gamble G.D. (2004). Factors Influencing Mortality in Acromegaly. J. Clin. Endocrinol. Metab..

[35] Dal J., Leisner M.Z., Hermansen K., Farkas D.K., Bengtsen M., Kistorp C., Nielsen E.H., Andersen M., Feldt-Rasmussen U., Dekkers O.M. (2018). Cancer Incidence in Patients with Acromegaly: A Cohort Study and Meta-Analysis of the Literature. J. Clin. Endocrinol. Metab..

[36] Falch C.M., Olarescu N.C., Bollerslev J., Dekkers O.M., Heck A. (2023). Trends in incidence and mortality risk for acromegaly in Norway: A cohort study. Endocrine.

[37] Cozzi R., Ambrosio M.R., Attanasio R., Bozzao A., De Marinis L., De Menis E., Guastamacchia E., Lania A., Lasio G., Logoluso F. (2020). Italian Association of Clinical Endocrinologists (AME) and Italian AACE Chapter Position Statement for Clinical Practice: Acromegaly—Part 2: Therapeutic Issues. Endocr. Metab. Immune Disord. Drug Targets.

[38] Xiao T., Jiao R., Yang S., Wang Y., Bai X., Zhou J., Li R., Wang L., Yang H., Yao Y. (2023). Incidence and risk factors of cancers in acromegaly: A Chinese single-center retrospective study. Endocrine.

[39] Fleseriu M., Biller B.M.K., Freda P.U., Gadelha M.R., Giustina A., Katznelson L., Molitch M.E., Samson S.L., Strasburger C.J., van der Lely A.J. (2021). A Pituitary Society update to acromegaly management guidelines. Pituitary.

[40] Demir A.N., Sulu C., Kara Z., Sahin S., Ozaydin D., Sonmez O., Keskin F.E., Tanriover N., Gazioglu N., Kadioglu P. (2023). Changing presentation of acromegaly in half a century: A single-center experience. Pituitary.

[41] Bałdys-Waligórska A., Krzentowska A., Gołkowski F., Sokołowski G., Hubalewska-Dydejczyk A. (2010). The prevalence of benign and malignant neoplasms in acromegalic patients. Pol. J. Endocrinol..

[42] Terzolo M., Reimondo G., Berchialla P., Ferrante E., Malchiodi E., De Marinis L., Pivonello R., Grottoli S., Losa M., Cannavo S. (2017). Acromegaly is associated with increased cancer risk: A survey in Italy. Endocr. Relat. Cancer.

[43] Ganokroj P., Sunthornyothin S., Siwanuwatn R., Chantra K., Buranasupkajorn P., Suwanwalaikorn S., Snabboon T. (2021). Clinical characteristics and treatment outcomes in acromegaly, a retrospective single-center case series from Thailand. Pan Afr. Med. J..

[44] Can M., Kocabaş M., Çordan İ., Çalişkan Burgucu H., Karaköse M., Kulaksizoğlu M., Karakurt F. (2021). Prevalence of comorbidities and associated factors in acromegaly patients in the Turkish population. Turk. J. Med. Sci..

[45] Jenkins P.J., Fairclough P.D. (2002). Screening guidelines for colorectal cancer and polyps in patients with acromegaly. Gut.

[46] Dworakowska D., Grossman A.B. (2019). Colonic Cancer and Acromegaly. Front. Endocrinol..

[47] Iwamuro M., Yasuda M., Hasegawa K., Fujisawa S., Ogura-Ochi K., Sugihara Y., Harada K., Hiraoka S., Okada H., Otsuka F. (2018). Colonoscopy examination requires a longer time in patients with acromegaly than in other individuals. Endocr. J..

[48] Kurimoto M., Fukuda I., Hizuka N., Takano K. (2008). The Prevalence of Benign and Malignant Tumors in Patients with Acromegaly at a Single Institute. Endocr. J..

[49] Chesnokova V., Zonis S., Zhou C., Recouvreux M.V., Ben-Shlomo A., Araki T., Barrett R., Workman M., Wawrowsky K., Ljubimov V.A. (2016). Growth hormone is permissive for neoplastic colon growth. Proc. Natl. Acad. Sci. USA.

[50] Mercado M., Gonzalez B., Vargas G., Ramirez C., de los Monteros A.L.E., Sosa E., Jervis P., Roldan P., Mendoza V., López-Félix B. (2014). Successful Mortality Reduction and Control of Comorbidities in Patients with Acromegaly Followed at a Highly Specialized Multidisciplinary Clinic. J. Clin. Endocrinol. Metab..

[51] Ljubičić N., Poropat G., Antoljak N., Marković N.B., Šakić V.A., Rađa M., Soldo D., Štimac D., Kalauz M., Iveković H. (2021). Opportunistic screening for colorectal cancer in high-risk patients in family medicine practices in the Republic of Croatia. Acta Clin. Croat..

[52] Woliński K., Stangierski A., Gurgul E., Bromińska B., Czarnywojtek A., Lodyga M., Ruchała M. (2017). Thyroid lesions in patients with acromegaly—Case-control study and update to the meta-Analysis. Endokrynol. Pol..

[53] Kan S., Kizilgul M., Celik B., Beysel S., Caliskan M., Apaydin M., Ucan B., Cakal E. (2019). The effect of disease activity on thyroid nodules in patients with acromegaly. Endocr. J..

[54] Xu D., Wu B., Li X., Cheng Y., Chen D., Fang Y., Du Q., Chen Z., Wang X. (2019). Evaluation of the thyroid characteristics of patients with growth hormone-secreting adenomas. BMC Endocr. Disord..

[55] Wu X., Gao L., Guo X., Wang Q., Wang Z., Lian W., Liu W., Sun J., Xing B. (2018). GH, IGF-1, and Age Are Important Contributors to Thyroid Abnormalities in Patients with Acromegaly. Int. J. Endocrinol..

[56] Dogansen S.C., Salmaslioglu A., Yalin G.Y., Tanrikulu S., Yarman S. (2019). Evaluation of the natural course of thyroid nodules in patients with acromegaly. Pituitary.

[57] Akhanli P., Hepşen S., Uçan B., Düğer H., Bostan H., Kizilgül M., Sencar M.E., Çakal E. (2021). The evaluation of breast findings detected through different visualisation techniques in acromegaly patients—A retrospective study. Turk. J. Med. Sci..

[58] Ruchala M., Skiba A., Gurgul E., Uruski P., Wasko R., Sowinski J. (2009). The occurrence of thyroid focal lesions and a need for fine needle aspiration biopsy in patients with acromegaly due to an increased risk of thyroid cancer. Neuro. Endocrinol. Lett..

[59] Bolanowski M., Zatonska K., Kaluzny M., Zielinski G., Bednarek-Tupikowska G., Bohdanowicz-Pawlak A., Daroszewski J., Szymczak J., Podgorski J.K. (2006). A follow-up of 130 patients with acromegaly in a single centre. Neuro. Endocrinol. Lett..

[60] Terzolo M., Puglisi S., Reimondo G., Dimopoulou C., Stalla G.K. (2020). Thyroid and colorectal cancer screening in acromegaly patients: Should it be different from that in the general population?. Eur. J. Endocrinol..

[61] Durmuş E.T., Atmaca A., Çolak R., Durmuş B. (2022). Cancer prevalence and cancer screening in patients with acromegaly: A single center experience. Endocrine.

